# Effect of CatSper and Hv1 Channel Inhibition on Progesterone Stimulated Human Sperm

**Published:** 2018

**Authors:** Sara Keshtgar, Hamideh Ghanbari, Esmaeel Ghani, Seyed Mostafa Shid Moosavi

**Affiliations:** 1- Department of Physiology, School of Medicine, Shiraz University of Medical Sciences, Shiraz, Iran; 2- Department of Physiology, Faculty of Medicine, Hormozgan University of Medical Sciences, Bandar Abbas, Iran; 3- Endocrinology and Metabolism Research Center, Hormozgan University of Medical Sciences, Bandar Abbas, Iran

**Keywords:** Acrosome reaction, CatSper channel, Hv1 channel, Progesterone, Sperm motility

## Abstract

**Background:**

Intracellular calcium and proton concentrations are important factors for activating human sperm. Calcium ion (Ca^2+^) enters sperm through voltage-dependent calcium channel of sperm (CatSper). Proton was extruded from sperm through voltage-gated proton channel (Hv1). In the present study, the selective inhibitors of the CatSper and Hv1 channels, NNC 55-0396 (NNC) and zinc ion, respectively, were used to investigate functions of these channels.

**Methods:**

Normal semen samples (n=24) were washed and diluted to 20×10^6^ *sperm/ml*. The diluted sample was divided into 8 groups, containing Ham’s F-10 (the control group), 2 *μM* NNC, 1 *mM* ZnCl_2_ and NNC+Zn. The other 4 groups were the same as above, except that they contained 1 *μM* progesterone. The computer assisted analysis was done by VT-Sperm 3.1 to determine the percentage of motile sperm and sperm velocity. Acrosomal status was monitored by FITC-PSA and viability assessed by Eosin–Y staining. Statistical comparisons were made using ANOVA followed by Tukey post hoc test. The p<0.05 was considered significant.

**Results:**

The percentage of viable and motile sperm, curvilinear velocity and other parameters of motility was reduced in all groups containing NNC, zinc and NNC+ zinc. Progesterone–induced acrosome reaction was abolished by each of these inhibitors. The combination effect of NNC plus zinc on motility and progesterone–induced acrosome reaction was not stronger than NNC by itself.

**Conclusion:**

CatSper and Hv1 channels play a critical role in human sperm function and viability. It seems that a functional relationship exists between CatSper and Hv1 channels.

## Introduction

Sperm hyperactivation is essential for fertilization and it occurs after incrementing calcium ion (Ca^2+^) in the flagellar part of sperm (1). Cytoplasmic Ca^2+^ has intracellular and extracellular sources. The stored Ca^2+^ in the cell can be liberated as a result of adequate stimuli (2, 3). Thimerosal, as a Ca^2+^ mobilizing agent from intracellular store, induces human sperm hyperactiveation (4). Voltage-gated calcium, transient receptor potential channels and CatSper channel are found in the sperm, and when they are appropriately stimulated, Ca^2+^ enters the sperm (5–8). CatSper is a sperm specific voltage-sensitive calcium channel, which is mainly located at the flagellar part of the sperm (9). Increase in intracellular pH is a main activator of CatSper channel (9), and it has been shown that alkalization of bovine sperm by NH_4_Cl causes its hyperactivation via opening of CatSper channel (10).

Flagellum of human sperm contains voltage-gated proton channel (Hv1), as well, which is responsible for proton exit from the cell and intracellular alkalization (11). Hv1 channel is a dimer and each subunit acts as a voltage-dependent proton channel (12). Intracellular pH is an important factor for controlling sperm motility (10, 13), acrosome reaction, and egg fertilization (14).

The most important Hv1 activating factors are membrane depolarization, extracellular alkalization and anandamide. Zinc (Zn) is the most potent inhibitory factor for Hv1 channel activity (11, 15). Outward current of proton from human spermatozoa was recorded with patch clamp technique. Proton current is inhibited by Zn dose dependently. Fifty percent of inhibition (IC50) was achieved with 222±36 *nM* of Zn and 1 *mM* of Zn completely inhibited the proton current. At the same conditions, other divalent cations such as calcium, barium, and magnesium even at millimolar concentrations had only a little effect on current through HV1 (11).

Moreover, progesterone plays an important role in human sperm activation. This steroid hormone is found in female genital tract and is released by cumulus cells surrounded the oocyte. Progesterone affects many important aspects of sperm physiology such as motility, acrosomal reaction, and chemotaxis (16). These effects of progesterone on sperm are fast and non–genomic, through increment of cAMP, intracellular calcium, and promotion of tyrosine phosphorylation of proteins (16). Progesterone induces Ca^2+^ influx into the spermatozoa and intracellular alkalization potentiates this effect (17). Some studies proved that progesterone-induced Ca^2+^ signals through CatSper in human spermatozoa reach the plateau at 1 *μM* of progesterone (17, 27). CatSper current in the presence of varying concentrations of progesterone showed that the amplitude of human CatSper current remained stable at 1 *μM* of progesterone (17) and maximum current is evoked by 1 *μM* of progesterone (27).

Patch clamp recording has shown that a T–type voltage-gated Ca^2+^ channel inhibitor, NNC55–0396 (NNC), inhibited the progesterone-activated current (17). It has been shown that calcium current through human CatSper was completely blocked by 2 *μM* of NNC (17). They also showed that progesterone does not have any significant effect on Hv1 channel activity (17). However, an agent such as 4–aminopyridine raises intracellular pH and mobilizes stored Ca^2+^ and leads to sperm hyperactivation (18).

Both CatSper and Hv1 are located within the same flagellar compartment of sperm (15). Regarding the channels’ characteristics, it was hypothesized that the function of these channels can be related to each other, especially whenever the sperm is stimulated by progesterone. Hence, to investigate this hypothesis, sperm motility, viability and acrosome reaction were assessed in a medium containing progesterone, when CatSper or Hv1 channels or both of them were blocked by their inhibitors.

## Methods

### Semen samples and sperm isolation:

Human semen samples (n=24) were obtained from healthy men (20–40 years old), who referred to Shiraz Infertility Centre. This study was done from October 2015 until September 2016. Donors were selected among normozoospermic non-smoking men who did not have any medical problems and had not used any drug, dietary supplements, and alcohol. The study protocol was approved by the research ethic committee of Shiraz University of Medical Sciences (Ethical Code: EC–92–6773). Semen samples were collected after at least 3 days of sexual abstinence. Total semen volume, pH and appearance were determined after liquefactions and sperm concentration, and motility was assessed by SQA–V^TM^ Sperm Quality Analyzer, Austria. After semen analysis, the remaining normozoospermic samples were used for aims of this study. The initial characteristics of selected semen samples (before washing) are reported in table 1. Semen samples were washed and the experiments were performed on swimming-up sperm. Samples were diluted to 20×10^6^ *sperm/ml* by Ham’s F10 and thoroughly mixed before aliquots were taken for assessment. One group was considered as control group containing only the sperm medium (Ham’s F10), NNC and Zn groups contained NNC and ZnCl_2_ solution with final concentration of 2 *μM* and 1 *mM*, respectively. Another group contained both NNC and Zn with the above mentioned concentrations. Other four groups were the same as above groups except that all of them contained 1 *μM* progesterone. Afterwards, the samples were incubated at 37°*C* and 5% CO_2_ for 30 *min*. At the end of the experiment, motility, parameters of motility, viability, and acrosome reaction of 24 samples in all groups were assessed.

**Table 1.** The characteristics of selected semen samples, before washing

**Semen characteristics**	**Mean±SE (n=24)**
**Semen volume (*ml*)**	4.3±0.2
**pH**	7.5±0.2
**Sperm concentration (×10^6^/*ml*)**	105.6±5.7
**Total sperm count (×10^6^)**	454.1±31.2
**Progressive motility (%)**	45.1±0.9
**Non–progressive motility (%)**	6.1±0.3
**Total sperm motility (%)**	51.2±0.6
**Immotile sperm (%)**	46.8±0.8
**Viability (%)**	56.6±2.2
**Normal morphology (%)**	52.9±1.2
**Viscosity**	Normal

### Chemical preparation:

One milligram of NNC 55-0396 (N0287, Sigma Aldrich, Germany) was dissolved in 1 *ml* deionized water to prepare stock solution with 1800 *μM* concentration. Aliquots were preserved at −20°*C* until usage.

ZnCl_2_ powder (Z0152, Sigma Aldrich, Germany) was dissolved in deionized water to prepare 73 *mM* stock solution, pH was evaluated and adjusted to 7.4. Then it was preserved at +4°*C* until usage.

Stock solution of progesterone (P8783, Sigma Aldrich, Germany) dissolved in ethanol (600 *μM*) was prepared and preserved at −20°*C*. The final concentration of ethanol in sperm medium was 0.1%.

### Sperm motility assessment:

After incubation, sperm motility was evaluated by sperm motility analyzer (VT–Sperm 3.1). For this purpose, the samples were mixed well and 10 *μl* of each sample was placed on a glass slide and covered with 22 *mm*× 22 *mm* cover slip. At least 10 individual fields were observed randomly under ×200 resolution. Average path velocity (VAP, *μm/s*), straight line velocity (VSL, *μm/s*), curvilinear velocity (VCL, *μm/s*), linearity (LIN, %), and percentages of sperm with progressive and non–progressive motility were recorded. A minimum of 200 cells were analyzed for each aliquot.

### Evaluation of acrosome reaction and cell viability:

Acrosomal status was monitored with acrosome specific FITC-PSA (Lectin from Pisum sativum, L0770, Sigma Aldrich, Germany) which was dissolved in PBS. Briefly, 10 *μl* from each group was smeared and fixed. Then slides were stained with 1 *mg/ml* FITC for 30 *min* at room temperature in dark. Then 10 *μg/ml* DAPI (D9542, Sigma Aldrich, Germany) was dissolved in DMSO and applied to the slides for 5 *min* at room temperature. With DAPI staining, the nucleus became dark blue and cells were recognizable easily under a fluorescent microscope (Olympus BX51, Japan). The cells were observed and photographed (19). The green staining fluorescent could be observed in the acrosomal area, if the acrosome was intact, and bright fluorescent staining was limited to the equatorial segment area, if it reacted.

Sperm viability was assessed by Eosin Y (E6003, Sigma Aldrich, Germany) staining method. Performing the experiment, equal amount of sample and Eosin Y were mixed thoroughly. Then 10 *μl* of that was smeared on the slide and after 30 *s* at least 200 cells were evaluated by an optical microscope (Olympus CX41, Japan) at magnification×200. The living cells were unstained whereas the dead ones became red.

### Statistical analysis:

Data was analyzed by SPSS 16.0 software. The results were expressed as mean±SE and p<0.05 was considered significant. Normal distribution of data was assessed by Kolmogorow–Smirnov test. Data were normally distributed. Therefore, statistical comparison between groups containing progesterone with related non-progesterone groups was done by paired t-test, and statistical analysis between all groups without or with progesterone were done by one-way ANOVA followed by Tukey post hoc test.

### Ethical approval:

All procedures performed in studies involving human participants were in accordance with the ethical standards of Shiraz University of Medical Sciences (Ethical Code: EC–92–6773) and with the 1964 Helsinki declaration and its later amendments or comparable ethical standards.

## Results

**Figure 1.**
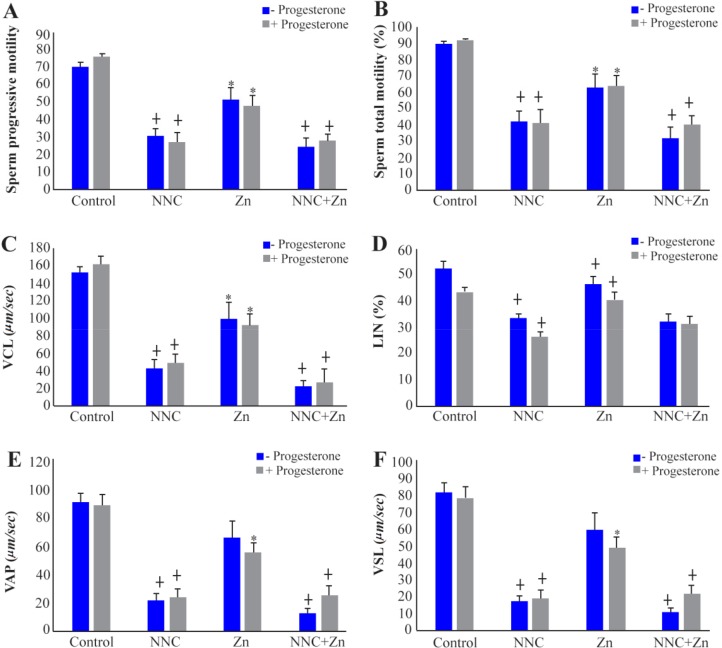
Effects of 1 *μM* progesterone, CatSper and Hv1 channel inhibitors (2 *μM* NNC and 1 *mM* Zn, respectively) on sperm progressive motility (A), total motility (B), VCL (C), LIN (D), VAP (E), and VSL (F). * Significant difference with control group, ┼ Significant difference with other groups (p<0.05)

CatSper and Hv1 channel inhibitors significantly decreased progressive and total motility. The highest decline in motility was observed in groups containing NNC. A significant difference in progressive motility was seen between NNC group and control group (p=0.000), as well as, NNC group and Zn group (p=0.003). The total motility was significantly decreased between NNC group and control group (p=0.000) and between NNC group and Zn group (p=0.012) (Figure 1, A and B).

Sperm VCL, VSL, VAP, and LIN were reduced significantly in the medium that contained NNC or NNC+Zn. Hv1 inhibitor channel, Zn, decreased sperm VCL (p=0.002), but its inhibitory effect on VAP and VSL appeared only in the medium that contained progesterone (Figure 1, C–F).

The percentage of acrosome reacted sperm significantly increased by progesterone (p=0.000) which was inhibited by NNC, Zn or NNC+Zn (Table 2).

**Table 2.** Effects of progesterone, CatSper and Hv1 channel inhibitors on sperm acrosomal reaction

**Subgroups (n=24)**	**Acrosome reacted sperm (%)**			**p**

	**Control**	**Progesterone (1 *μM*)**		
**Ham’s F10**	7.1±0.8	21.9±1.7		p<0.001^†^
**NNC (2 *μM*)**	7.1±0.8	7.7±0.8	p<0.001^*^	p=0.60^†^
**Zn (1 *mM*)**	8.9±0.7	9.8±4.7	p<0.001^*^	p=0.42^†^
**NNC + Zn**	8.4±0.7	7±0. 6	p<0.001^*^	p=0.23^†^

Statistical data are represented as mean±SEM.

* Representative comparison between subgroups of control group,

† Representative comparison between control and progesterone containing subgroups

Inhibition of CatSper and Hv1 channels increased sperm mortality (Table 3). Highest mortality rate was seen when both CatSper and Hv1 channels were concurrently inhibited in the presence of progesterone (p<0.001).

**Table 3.** Effects of progesterone, CatSper and Hv1 channel inhibitors on sperm viability

**Subgroups (n=24)**	**Live sperm (%)**				**p**

	**Control**		**Progesterone (1 *μM*)**		
**Ham’s F10**	90±1.03		92±1.12		p=0.61^†^
**NNC (2 *μM*)**	73±2.14	p<0.001^*^	69±3.33	p<0.001^*^	p=0.36^†^
**Zn (1 *mM*)**	79.3±1.6	p=0.01^*^	75±2.22	p<0.001^*^	p=0.25^†^
**NNC + Zn**	65±4.47	p<0.001^*^	53.1±5.79	p<0.001^*^	p=0.004^†^

Statistical data are represented as mean± SEM.

* Representative comparison between subgroups of each control group or progesterone containing group,

† Representative comparison between control and progesterone containing subgroups

## Discussion

Sperm motility is an important factor for fertilization. Many factors affect sperm motility such as Ca^2+^, increased pH, cAMP signaling, and factors which exist in the female genital tract, especially progesterone (13). The effect of progesterone on human sperm motility and induction of sperm hypermotility is less clear and it is controversial (16). Some studies have shown the positive effect of progesterone on progressive motility and hyperactivation (20–24) while others observed that 1 *pM* and 10 *μM* of progesterone did not change sperm progressive motility (25, 26). However, progesterone could induce sperm chemotaxis towards oocyte and acrosomal reaction (16).

Assessment of progesterone-induced Ca^2+^ signals in human sperm showed that progesterone has an early and transient effect which is followed by a late very weak effect, but sustained on Ca^2+^ inward current (27). The dose response curve of progesterone versus inward current through CatSper channel showed that this current reaches to a plateau at about 1 *μM* (17). However, 3 *μM* progesterone did not induce hyperactivation (4). In our experiment, sperm was collected by swim-up method and all of the live sperm in the control and progesterone containing groups were motile after 30 *min* of incubation. Therefore, the percentage of the motile sperm did not show any increment in progesterone group in comparison with control group, though VCL increased non-significantly in the progesterone group.

Some studies have suggested that the inward Ca^2+^ current through the CatSper was augmented in the presence of progesterone and the Ca^2+^ current was blocked by 2 *μM* NNC (17, 27). In this study, NNC (2 *μM*) reduced total motility to approximately 47 to 50%. Progressive motility, VSL, VCL, and VAP were significantly decreased in all groups containing NNC (Figure 1). It has been shown that even higher concentration of NNC (10 and 20 *μM*) had the similar effect during 15 and 60 *min* of incubation (25).

It has been proved that hyperactivated motility of mouse sperm driven by CatSper is required for fertilization (28) and patients with genomic CatSper defect are likely sterile (29). Moreover, the CatSper expression decreased in asthenozoo-spermia in comparison with normozoospermia (25). Our results emphasized the importance of CatSper channel function in human sperm motility. Because NNC could not stop all of the motile sperm, it is suggested that in addition to Ca^2+^ current through CatSper channel, Ca_i_^2+^ might be provided by other sources. For example, it is possible that other factors such as cAMP, intracellular alkalization and tyrosine phosphorylation might control motility, as well (9, 18).

Intracellular alkalization strongly activates the CatSper channel (11). In human sperm, Hv1 channel is responsible for this alkalization (11). An attempt was made to find the importance of Hv1 channel in sperm motility and its functional relationship with CatSper. Hv1 inhibition by 1 *mM* Zn reduced the total motility percentage by 27%. In addition, motility parameters such as VCL and LIN were affected by Zn. The reduction in sperm motility did not change when CatSper and Hv1 were blocked concurrently, in comparison to only CatSper channel inhibition. Our result showed the important role of CatSper and Hv1 channels in sperm motility and parameters of motility, in control and progesterone-stimulated condition.

Increase in Ca_i_^2+^ and intracellular alkalization are important factors for sperm capacitation and acrosome reaction (15). Moreover, progesterone is another potent acrosome reaction inducer (22, 25). In this study, progesterone-stimulated acrosome reaction was inhibited by adding NNC and/or Zn. Inhibition of human sperm capacitation and acrosome reaction by Zn was previously reported (11). low levels of Hv1 mRNA were reported in some infertile patients (30). Moreover, it is proven that CatSper channel has a role in progesterone–induced acrosome reaction (25). Our results showed that CatSper and Hv1 channels have a significant role in progesterone–induced acrosome reaction. The combination effect of NNC+Zn on progesterone–induced acrosome reaction was not stronger than NNC or Zn alone. Regarding the sensitivity of CatSper to intracellular alkalization, it is most likely that blocking the Hv1 reduces acrosome reaction through inhibition of CatSper.

In our experiment, NNC and Zn reduced the percentage of viable sperm. In general, cell death and apoptosis are associated with Ca_i_^2+^ homeostasis (31). One study showed that NNC reduced MOLT–4 and Jurkat cell viability in a dose–dependent manner through the cell shrinkage, chromatic agglutination, apoptosis, and depolarization of the mitochondrial membrane potential (32). Inhibition of voltage-dependent K^+^ channels in smooth muscle of rabbit coronary arteries was also observed, in response to NNC (33). According to these findings, it is concluded that NNC induces sperm apoptosis, through its function as a Ca^2+^ channel blocker or other unknown pathways.

Hv1 channel plays a significant role in ionic regulation in human sperm and it is quite likely that blockage of the Hv1 channel affects viability of the human sperm. The highest reduction in the percentage of live sperm was observed in the group which contained progesterone, NNC and Zn. Actually, further research is required to determine the mechanism by which blocking Hv1 and CatSper channels affect human sperm viability. This study had some limitations; ion current through the channels was not investigated and the expression of CatSper and Hv1 channels was not assessed. However, our result suggested that any defect in CatSper and Hv1 channels can affect human sperm physiology and may lead to infertility. The clarification of exact intracellular mechanisms of these channels could be considered for treatment of infertile men.

## Conclusion

In conclusion, CatSper and Hv1 channels play a critical role in sperm function and viability, under control and stimulated conditions by progesterone. It was hypothesized that there is a functional relationship between CatSper and Hv1 channels, even in unstimulated condition. Therefore, Hv1 opening may lead to CatSper activity and Ca^2+^ entrance since CatSper is required for sperm motility, viability and acrosome reaction. The clarification of exact intracellular mechanisms needs further investigation.
